# Widespread underestimation of rain-induced soil carbon emissions from global drylands

**DOI:** 10.1038/s41561-025-01754-9

**Published:** 2025-07-31

**Authors:** Ngoc B. Nguyen, Mirco Migliavacca, Maoya Bassiouni, Dennis D. Baldocchi, Laureano A. Gherardi, Julia K. Green, Dario Papale, Markus Reichstein, Kai-Hendrik Cohrs, Alessandro Cescatti, Tuan Dung Nguyen, Hoang H. Nguyen, Quang Minh Nguyen, Trevor F. Keenan

**Affiliations:** 1 Department of Environmental Science, Policy, and Management, University of California, Berkeley, Berkeley, CA USA; 2 European Commission, Joint Research Centre, Ispra, Italy; 3 Department of Environmental Science, University of Arizona, Tucson, AZ USA; 4 National Research Council, Institute of Research on Terrestrial Ecosystems, Monterotondo Scalo, Italy; 5 University of Tuscia, DIBAF, Viterbo, Italy; 6 Max Planck Institute of Biogeochemistry, Department of Biogeochemical Integration, Jena, Germany; 7 Universitat de València, València, Spain; 8 Department of Computer and Information Science, University of Pennsylvania, Philadelphia, PA USA; 9 H. Milton Stewart School of Industrial and Systems Engineering, Georgia Institute of Technology, Atlanta, GA USA; 10 Department of Electrical Engineering and Computer Science, Massachusetts Institute of Technology, Cambridge, MA USA; 11 Climate and Ecosystem Sciences Division, Lawrence Berkeley National Laboratory, Berkeley, CA USA

**Keywords:** Ecosystem ecology, Carbon cycle

## Abstract

Dryland carbon fluxes, particularly those driven by ecosystem respiration, are highly sensitive to water availability and rain pulses. However, the magnitude of rain-induced carbon emissions remains unclear globally. Here we quantify the impact of rain-pulse events on the carbon balance of global drylands and characterize their spatiotemporal controls. Using eddy-covariance observations of carbon, water and energy fluxes from 34 dryland sites worldwide, we produce an inventory of over 1,800 manually identified rain-induced CO_2_ pulse events. Based on this inventory, a machine learning algorithm is developed to automatically detect rain-induced CO_2_ pulse events. Our findings show that existing partitioning methods underestimate ecosystem respiration and photosynthesis by up to 30% during rain-pulse events, which annually contribute 16.9 ± 2.8% of ecosystem respiration and 9.6 ± 2.2% of net ecosystem productivity. We show that the carbon loss intensity correlates most strongly with annual productivity, aridity and soil pH. Finally, we identify a universal decay rate of rain-induced CO_2_ pulses and use it to bias-correct respiration estimates. Our research highlights the importance of rain-induced carbon emissions for the carbon balance of global drylands and suggests that ecosystem models may largely underrepresent the influence of rain pulses on the carbon cycle of drylands.

## Abstract (web summary)

Eddy-covariance observations suggest that rain pulses over global drylands drive substantial soil carbon emissions, which are underestimated in current measurement and modelling approaches.

## Main

Drylands cover over a third of the global land surface^1,2^ and substantially influence the trend and interannual variability of the terrestrial carbon sink^3–5^. These ecosystems are water limited, with rainfall driving vegetation and microbial processes that impact ecosystem carbon dynamics^6^. Sporadic rain pulses in particular play a crucial role in determining plant growth, microbial activity, soil moisture and overall ecosystem productivity^7–9^. Although the influence of water availability on dryland carbon dynamics is well established^6,10,11^, the processes governing responses to rain pulses remained poorly understood. The amount of carbon lost due to heterotrophic respiration stimulated by rain-pulse events and the key drivers of those losses across diverse dryland ecosystems, remain open questions^12–15^, and the underlying processes are thus typically not included in models of dryland carbon dynamics^14,16,17^.

**Fig. 1:**
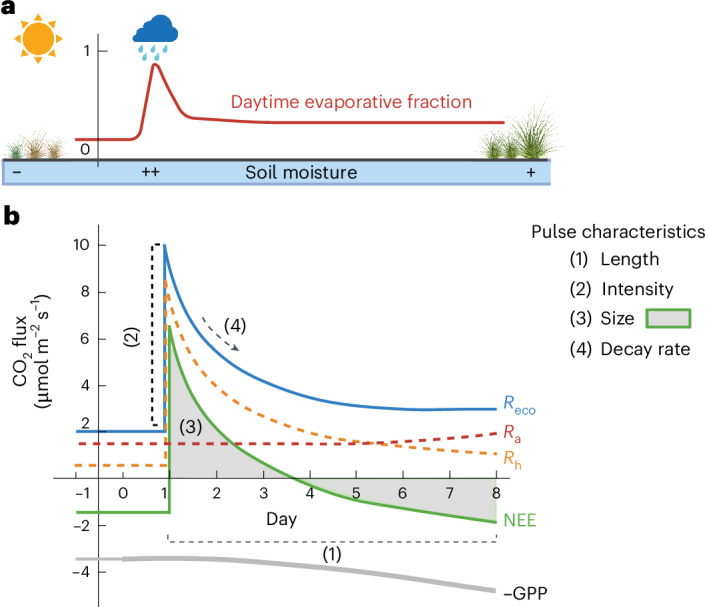
Conceptual diagram of pulse events. **a**, Pulse events, or rain-induced CO_2_ pulse events, are defined as ecological processes in which rainfall on previously dry soils triggers pulses of soil CO_2_ and, consequently, pulses of net CO_2_ flux (NEE). The horizontal bar represents the soil moisture gradient, daytime EF and vegetation growth before and after rain pulses. **b**, The dynamics of daily ecosystem carbon fluxes (*R*_a_, *R*_h_, *R*_eco_, NEE and GPP) during pulse events and CO_2_ pulse characteristics: (1) length, (2) intensity, (3) size and (4) decay rate (Methods). *R*_eco_ is ecosystem respiration, *R*_a_ is autotrophic respiration and *R*_h_ is heterotrophic respiration. The NEE of CO_2_ flux was measured directly from eddy-covariance towers. We plotted −GPP instead of GPP for visualization purposes. The first-principle relationship between carbon fluxes can be described as follows: NEE = *R*_a_ + *R*_h_ – GPP, *R*_eco_ = *R*_a_ + *R*_h_. Figure created with BioRender.com.

Rain pulses trigger abiotic and biotic soil CO_2_ pulses, a phenomenon often termed a rain-induced CO_2_ pulse event or pulse event. Abiotic CO_2_ pulses occur when water displaces CO_2_ from soil pores^18,19^ or dissolves carbonates^20^. Biotic CO_2_ pulses, known as the Birch effect, result from microbial respiration surges following the sudden availability of labile carbon and nutrients^13,21–24^. This resource pulse may arise from the release of intracellular solutes, microbial lysis and physical breakdown of soil aggregates^23,25,26^. While abiotic CO_2_ pulses are short lived^20,27^, Birch-effect CO_2_ pulses can last several days and lead to significant substantial ecosystem carbon losses during the day and night^13,15,28,29^. Microbial respiration can increase 60–80-fold after rainfall^15^, then decay as the soil dries^13–15,21,30^, contributing up to 40% to total soil respiration during the growing season^29^ and 5–10% to annual net ecosystem productivity (NEP)^28^ (Fig. 1). The intensity of these pulses has been linked to hydrologic dynamics such as the intensity/frequency of rewetting and antecedent soil dryness^13–15,18^. By contrast, plant–microbe interactions are often neglected, mainly because previous research focused on soil-centric environments or vegetation-senescence periods^12,20^. The combined effects of vegetation, soil properties, climate and hydrologic dynamics on rain-induced carbon losses, therefore, remains poorly constrained.

Modelling these pulse-driven events is particularly challenging due to the sporadic and discontinuous nature of rainfall in drylands^6^. Most models effectively incorporate steady-state processes such as responses to temperature, long-term changes in soil moisture and vegetation productivity. However they largely neglect non-steady-state but important rain-induced carbon losses^15,31–35^. This omission could lead to substantial underestimation of soil and ecosystem respiration, potentially resulting in inaccurate estimates of related processes such as gross primary productivity (GPP)^14,36^. The lack of a broad characterization of dryland rain-induced CO_2_ pulses has hindered the integration of these processes into existing ecosystem models.

Detecting rain-induced carbon losses across diverse dryland ecosystems is a critical first step to improving model predictions of dryland carbon fluxes. FLUXNET, which provides global eddy-covariance measurements of net CO_2_ flux or net ecosystem exchange (NEE)^37,38^, offers a unique opportunity to analyse CO_2_ pulse responses at high temporal resolution. Rainfall-driven ecosystem respiration (*R*_eco_) pulses manifest as net CO_2_ anomalies, because microbial activity typically responds more rapidly to rainfall than vegetation^6,11,26,31,39–41^ (Fig. 1). However, FLUXNET does not provide continuous measurements of soil and ecosystem respiration^36^, making NEE the variable of choice for detecting rain-induced carbon losses. Furthermore, conventional eddy-covariance partitioning methods estimate *R*_eco_ using an Arrhenius-type temperature function^34,42^ (equation (2)), which may not effectively capture rain-induced carbon losses^35,36^. NEE observations from FLUXNET thus provide a unique opportunity to both identify and characterize soil CO_2_ pulses.

Here, we combine a novel manually labelled database of 1,857 pulse events with machine learning frameworks to detect rain-induced carbon losses across 34 dryland eddy-covariance sites, encompassing 323 years of half-hourly observations. Using this database, we characterize the spatiotemporal drivers of net CO_2_ pulse dynamics and bias-correct *R*_eco_ estimates derived from the conventional night-time partitioning method. We refer to this integrated detection and correction approach as FluxPulse. Our results highlight widespread underestimation of carbon losses during rain-pulse events in global drylands, with important implications for dryland carbon balance variability under climate change.

## Widespread underestimation of pulse event carbon fluxes

**Fig. 2:**
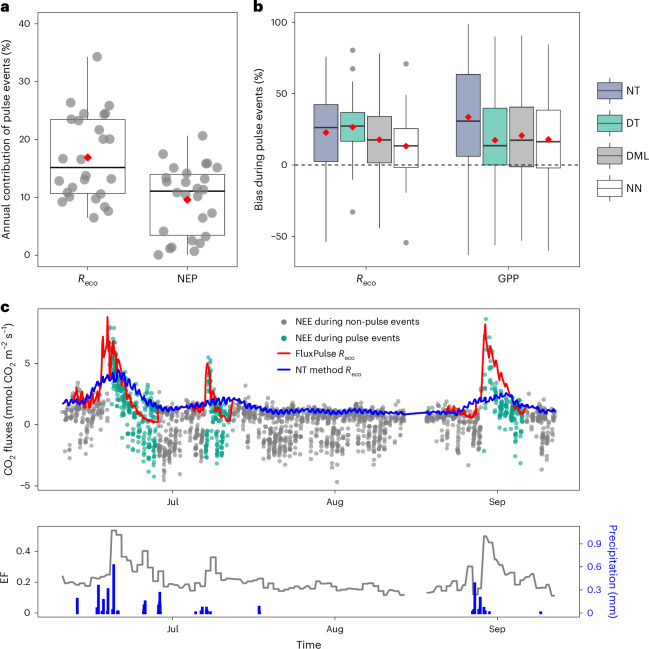
Partitioning methods underestimate pulse event CO_2_ fluxes. **a**, Contributions of ecosystem respiration (*R*_eco_) and NEP during pulse events to annual *R*_eco_ and NEP. Pulse event contribution (%) at each individual site is calculated as $\frac{{{\mathrm{sum}}}\left(x\right)}{{{\mathrm{sum}}}\left(\,y\right)}\times 100$, in which *x* is FluxPulse bias-corrected half-hourly *R*_eco_ or NEP during pulse events, and *y* is all available half-hourly bias-corrected *R*_eco_ or NEP (two sites whose NEP contribution values greater than three times of standard deviation were removed in the boxplots for visualization purposes). **b**, Performance of partitioning methods on estimating ecosystem respiration (*R*_eco_) and GPP during pulse events. The fluxes estimated from four methods were compared with the FluxPulse bias-corrected *R*_eco_ and GPP, which are the night-time (NT) method, the daytime method (DT), DML and NN (Methods). The flux underestimation is calculated as $\frac{{{\mathrm{sum}}}\left(x\right)-{{\mathrm{sum}}}(\;y)}{{{\mathrm{sum}}}\left(x\right)}\times 100$, in which *x* is the FluxPulse half-hourly *R*_eco_ or GPP and *y*is the partitioned half-hourly *R*_eco_ or GPP from different partitioning methods. Thus, the positive *y*values indicate flux underestimations, while negative *y*values indicate flux overestimations. In **a** and **b**, 28/34 sites whose FluxPulse significantly reduces biases (*P* < 0.001) (Extended Data Fig. 6) are included. The boxes represent interquartile range (IQR) marked with 25th, 50th and 75th percentile and whiskers extending to 1.5 times the IQR. The red diamonds represent the mean of the boxplots. In **b**, all the boxplots have the mean of distribution statistically greater than 0 (one-tailed paired *t*-test, *P* < 0.01). **c**, Half-hourly time series visualization of NEE, ecosystem respiration estimated by the FluxPulse bias-corrected algorithm (FluxPulse *R*_eco_) and by the NT method *R*_eco_ at the Albuera site, Spain (ES-Abr) in 2017. The NEE points corresponding to pulse events are manually labelled in green, while others are labelled in black. The red line represents FluxPulse bias-corrected *R*_eco_, and the blue line represents *R*_eco_ estimated by the NT method. In the lower graph, the black line shows the daytime EF, calculated daily. The blue bars are precipitation downscaled from ERA-Interim reanalysis data products, with half-hourly resolution. A sharp rise in EF and increased precipitation indicates the occurrence of pulse events and net CO_2_ pulses. Source data

We found that pulse events greatly impact the dryland carbon balance, contributing 16.9% ± 2.8% (mean ± the margin of error) to annual ecosystem respiration (*R*_eco_) and 9.6% ± 2.2% to NEP across sites (Fig. 2a). These findings align with previous research showing that soil CO_2_ pulses contribute 5–10% of annual NEP in a mid-latitude forest, with carbon losses during pulse events being comparable with the annual NEP of many ecosystems^15,28^.

Despite this substantial contribution, we found that all four existing eddy-covariance partitioning methods significantly underestimate *R*_eco_ and GPP during the pulse events when compared our FluxPulse bias-corrected fluxes (Fig. 2b). Underestimation for *R*_eco_ ranged from 13.4% ± 9.4% to 26.7% ± 9.0%, while GPP ranged from 17.8% ± 11.7% to 33.8% ± 14.2% (Fig. 2b,c). Since GPP is estimated as the difference between *R*_eco_ and NEE, any underestimation of *R*_eco_ directly affects the GPP estimates (Fig. 1).

The four partitioning methods examined include parametric approaches (night-time method^34^ and daytime method^42^) and machine learning methods (double machine learning (DML)^43^ and artificial neural networks (NN)^44^) (Methods). We performed a one-tailed paired *t*-test to compare partitioning methods, which revealed a clear ranking of *R*_eco_ underestimation during pulse events, with: NN (13.4% ± 9.4%) < DML (17.9% ± 9.7%) < night-time (22.9% ± 10.7%), daytime method (26.7% ± 9.0%) (*P* < 0.01) (Fig. 2b). The NN approach underestimates *R*_eco_ the least, followed by the DML approach, while the night-time and daytime methods exhibit similar underestimations. Regarding GPP during pulse events, the night-time method shows the largest bias (*P* < 0.01): night-time (33.8% ± 14.2%), daytime (17.8% ± 11.7%), DML (20.9% ± 12.4%) and NN (18.2% ± 11.2%) (Fig. 2b).

Among the four partitioning methods assessed, machine learning approaches (NN and DML) underestimate *R*_eco_ the least, highlighting their improved performance at capturing non-steady-state events compared with the parametric approaches assessed^44^. The underestimation of *R*_eco_ also leads to underestimation of GPP, particularly in methods where GPP is calculated as *R*_eco_ minus NEE. The night-time method shows the largest GPP underestimations (Fig. 2b), often leading to systematic negative half-hourly *GPP* during the first few days of pulse events^36^.

Actual biases in existing methods for estimating *R*_eco_ and GPP during pulse events could be much higher than those reported here. Our approach assumes minimal GPP during the first day of pulse events, using maximum NEE to estimate maximum *R*_eco_, given that the GPP estimates are not reliable during pulse events (Methods). Minimal GPP at the beginning of pulse events could happen in some sites due to the intensive prepulse dryness; however, we demonstrate that this is not the case in most sites (Extended Data Fig. 7). Even though the underestimates we quantified are conservative, our FluxPulse bias-correction method significantly reduces biases compared with existing partitioning methods, providing more reliable estimates of carbon fluxes during pulse events.

## Convergence in decay rate of rain-induced carbon pulses

**Fig. 3:**
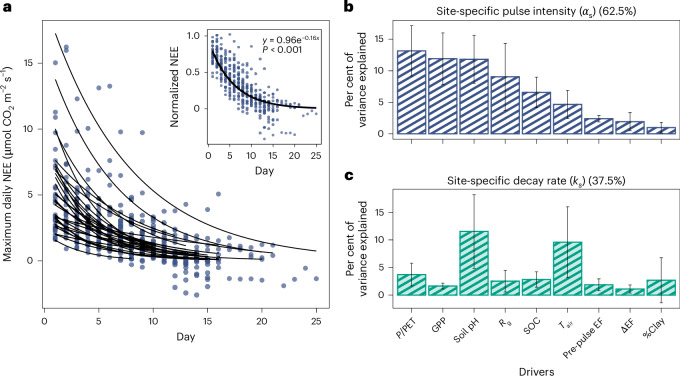
Rain-induced CO_2_ pulses decay across sites. **a**, NEE declines over time during pulse events across 29/34 sites with statistically significant site-specific decay rate (*P* < 0.05). The *P*value is derived from the statistical test for parameter estimates when fitting the decay function to the data. The *y* axis of the outer graph is the maximum of daily mean NEE from all pulse events from one site, and the x axis is the number of days after the pulse event starts, assuming that rainfall starts on day 1. The first-order kinetic reaction function is fitted for each site and displayed as black lines (equation (2)). The inset presents the same data but normalized by site-specific pulse intensity (${\alpha }_{{{s}}}$) and reveals a convergent decay rate across sites (*k*_*s*_ = 0.16 ± 0.01, *P* < 0.001). **b**,**c**, A relative importance analysis of nine predictors for site-specific pulse intensity (${\alpha }_{{{s}}}$) (**b**) and decay rate (*k*_*s*_) (**c**) across 29/34 sites. The predictors related to soil characteristics are clay percentage (%Clay), SOC from 0 to 5 cm (g kg^−1^) and soil pH. The predictor representing vegetation productivity is annual GPP obtained from the night-time (NT) partitioning method (µmol CO_2_ m^−2^ s^−1^). The predictors representing hydrologic conditions are aridity index (*P*/PET), rewetting intensity (ΔEF) and antecedent water availability (prepulse EF). The predictors representing climatic conditions are global shortwave radiation (*R*_g_) (W m^−2^) and air temperature (*T*_air_) (^o^C). The data in the bar plots are presented as the mean value ± standard deviation. The standard deviation of the bars is calculated by bootstrap sampling (100 times resampling with replacement). All predictors explain 62.5% and 37.5% of the variance in the site-specific pulse intensity and decay rate, respectively. Source data

Analysing more than 1,800 pulse events, we found that rain-induced carbon pulses decay exponentially after rain events in 85% of sites, following a universal exponential decay pattern (Fig. 3a). The intensity and decay rates of these pulses are primarily governed by environmental factors related to vegetation growth, climate and soil properties (Fig. 3b,c). We calculated site-specific pulse intensity (${\alpha }_{{{s}}}$) and decay rate (*k*_*s*_) by fitting a first-order kinetic reaction function to the daily maximum NEE across all pulse events at each site (Fig. 3a). The site-specific pulse intensity represents the maximum magnitude of rain-induced carbon losses at a site, and the site-specific decay rate represents how fast the carbon losses decay over time (equation (1)).

When normalizing the NEE rain-pulse response curves by site-specific pulse intensity, the response curves across sites converge to a universal decaying function ($y=0.96\times {{\mathrm{e}}}^{-0.16x}$) (Fig. 3a). This indicates a consistent response of soil microbes to rain pulses, probably due to the strong relationship between soil microbes, soil water potential and evaporation^45^. Prior studies have shown that cumulative soil evaporation follows a square root of time as the soil dries^46^, further supporting this convergence. Our finding of the convergent decay rate could facilitate the future incorporation of the rain-pulse effect into large-scale ecosystem models. The decaying pattern of net CO_2_ flux over time probably results from resource depletion (labile carbon, nitrogen, water availability and so on), changes in microbial community composition^26^ and vegetation growth triggered by pulse events which off-sets CO_2_ release. The widespread decay pattern highlights the biological influence of rainfall on ecosystem processes beyond abiotic influences, reinforcing the importance of the Birch effect in dryland carbon cycling. Our findings align with previous regional and laboratory studies^13,15,21,47^, confirming the widespread occurrence of the Birch effect in global drylands.

**Fig. 4:**
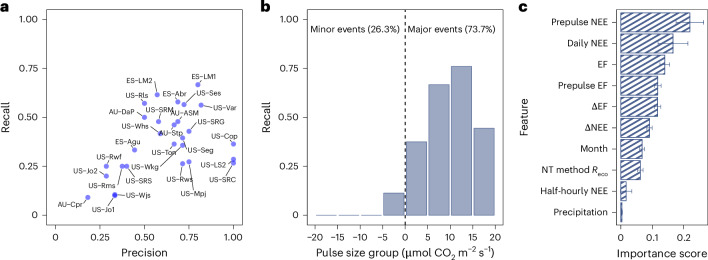
Random forest performance for detecting pulse events. **a**, A performance for detecting the start date of pulse events (within ±2 days error) across 28 qualified sites (see Supplementary Table 1 for more details). Precision is the fraction of pulse events the model detected that were real, while recall is the fraction of actual pulse events that were successfully detected by the model. **b**, The recall rate for different pulse size groups (divided into equal intervals of 5 µmol CO_2_ m^−2^ s^−1^, ranging from −20 to 20 µmol CO_2_ m^−2^ s^−1^). The dashed line *x* = 0 defines major (*x* > 0) and minor (*x* < 0) pulse events based on the size of net CO_2_ pulses. Major pulse events (73.7% of the testing set) are defined as carbon sources, while minor pulse events (26.3%) are carbon sinks. **c**, Feature importance score for all variables used in the random forest model to detect pulse events across 28 sites. The data are presented as the mean value ± standard deviation of accumulation of the impurity decrease for each feature within each decision tree. Source data

Multiple environmental factors influence the spatial dynamics of net carbon fluxes during pulse events. The three strongest predictors for the site-specific pulse intensity (${\alpha }_{{{s}}}$) across sites are aridity index (*P*/PET), GPP and soil pH, together with other factors explaining 62.5% variance (Fig. 3b). ${\alpha }_{{{s}}}$ increases with Aridity index (*P*/PET) and GPP but decreases with soil pH (Extended Data Fig. 9). For the site-specific decay rate (*k*_*s*_), soil pH and air temperature were important predictors, with *k*_*s*_increasing with soil pH and temperature (Extended Data Fig. 8). However, compared with ${\alpha }_{{\rm{s}}}$, environmental factors explain only 37.5% of variance in *k*_*s*_(Fig. 3b,c). Regarding hydrologic factors, we used evaporative fraction (EF), the fraction of available energy allocated to evapotranspiration, as a proxy of soil water availability and rainfall occurrence (Methods). Despite exhibiting a strong temporal correlation with pulse intensity and size, prepulse EF (the 14-day average EF before a pulse event) and ΔEF (a measure of the rewetting intensity) do not significantly explain the spatial variability of net CO_2_ pulses during pulse events (Figs. 3b,c and 4c and Extended Data Fig. 4).

Our results show that vegetation productivity is key to initial rewetting responses, as more productive sites supply more labile carbon through root exudation and plant litter, enhancing microbial decomposition^12,15,48^ (Fig. 3b,c). This also explains why soil organic carbon (SOC) shows little explanatory power for the spatial distribution of pulse characteristics (Fig. 3b,c), since SOC composition (for example, labile versus recalcitrant SOC) is likely more important than total SOC in sustaining the Birch effect^23,49^ (Fig. 3b,c). Besides GPP, the aridity index (*P*/PET) is a major predictor of pulse intensity across sites (Fig. 3b). Among all dry sites, wetter sites have higher pulse intensity (Extended Data Fig. 9). This suggests that wetter dryland sites have sufficient moisture to enhance vegetation growth, supplying substrates for microbial decomposition, while they are still dry enough for the Birch effect to occur.

Among all environmental factors, soil pH is a top predictor of both site-specific pulse intensity and decay rate (Fig. 3b,c). Previous studies have highlighted the influence of soil pH on microbial composition^50–56^, with soil microbial diversity peaks at near-neutral soil pH at a continental scale^53^. In our study, we also observe that the site-specific pulse intensity peaks at pH 6 and gradually declines as the pH increases to 8 (Extended Data Fig. 9). This could be attributed to various soil characteristics associated with soil pH, as well as physiological constraints on soil bacteria under extremely acidic or alkaline conditions^53^. Our validation of SoilGrids pH against site-specific measurements confirms soil pH measurement robustness (Extended Data Fig. 3). Our study shows that soil pH is an important spatial driver of rain-induced carbon pulses, potentially due to its influence on soil microbial dynamics.

## Detecting and characterizing rain-induced carbon pulses

Rain-induced CO_2_ pulse events are non-steady-state ecological processes, making them challenging to detect and model across sites. Nevertheless, automated pulse detection is now possible, given the large amount of data and a better understanding of the complexity of rain-pulse responses. We developed an automated detection framework using a random forest binary classification algorithm, in addition to manually labelling pulse events (Methods). Our results demonstrate that although performance varies across sites, which is possibly due to data quality and site-specific measurement uncertainties, such automated detection is applicable across diverse ecosystems. We successfully detected at least 60% of pulse events in 15/28 sites (Fig. 4a). Detection is most effective for major pulse events—those with net positive CO_2_ emissions, with 76% of pulses whose size between 10 and 15 µmol CO_2_ m^−2^ s^−1^ being correctly detected across sites (Fig. 4b).

We used two common classification metrics to evaluate model performance: precision and recall. Precision is the fraction of detected pulse events that are actual pulse events, while recall is the fraction of actual pulse events correctly detected by the model. Actual pulse events, in this case, are manually labelled pulse events. For example, if 12 pulse events occur, and the model detects 10 events, with only 7 of them being real, then precision is 7/10 (70%) and recall is 7/12 (58%) (Methods). Across sites, the model achieves a precision greater than 0.60 at 15/28 sites (Fig. 4a). Recall rates are generally lower than precision, with the highest recall rate (0.67) observed at the ES-LM1 site (Fig. 4a and Supplementary Table 1). Recall improves with the net CO_2_ pulse size, reaching 0.76 for pulses ranging from 10 to 15 µmol CO_2_ m^−2^ s^−1^ (Fig. 4b). Although the model did not effectively capture pulse events with negative pulse sizes, it performed well on most pulse events (73.7% of the pulse events in the testing set), particularly those with positive pulse sizes (Fig. 4b).

Among the features used for classification (Methods), prepulse NEE and daily mean NEE are the two most important predictors of the forest detection algorithm (Fig. 4c), meaning that ecosystem status before pulse events is crucial to detecting them. Pulse occurrence is more likely with positive prepulse NEE and less likely with negative prepulse NEE values when vegetation is active (Extended Data Fig. 10). In a woody savanna, the magnitude of CO_2_ pulses has also been shown to be inversely correlated with prepulse *R*_eco_(ref. ^15^).

Moreover, EF-derived indices (EF, antecedent water availability (prepulse EF) and rewetting intensity (ΔEF)) accurately describe temporal variability in pulse characteristics and improve machine learning detection performance (Figs. 3b,c and 4c and Extended Data Fig. 4). Lower prepulse EF and higher EF associate with a higher chance of pulse events (Extended Data Fig. 10). Furthermore, our analysis across multiple sites indicates that more intense rewetting events (ΔEF) and drier prepulse periods (prepulse EF) are linked with higher pulse intensity (ΔNEE) and greater pulse size (Extended Data Fig. 4). This indicates that larger pulses are more likely after prolonged dry periods followed by intense rewetting. Our findings suggest that under future scenarios of more intense precipitation and prolonged dry intervals, rain-induced carbon losses will probably intensify^57^.

Including site-static variables (for example, soil pH, vegetation productivity and aridity index) does not enhance model performance, though adding month as a temporal variable enhances results (Fig. 4c). Interestingly, precipitation is not a substantial predictor of pulse occurrence or intensity (Fig. 4c and Extended Data Fig. 4), most likely because EF already captures rainfall effects, and not all rainfall events trigger CO_2_ pulses.

Overall, EF is an integrated index that captures water dynamics, including the wetting intensity and antecedent water availability. Compared with precipitation, EF-derived indices play an important role in the pulse detection algorithm (Fig. 4c) and they significantly correlate with pulse intensity and size across most sites (Extended Data Fig. 4). While precipitation is undoubtedly a major driver of rain-induced CO₂ pulses, its direct influence is difficult to detect due to under-report rainfall from using the tipping bucket method and landscape heterogeneity^58,59^. EF, which integrates both wetting intensity and antecedent water availability, could thus be a promising index for water availability particularly at eddy-covariance sites where precipitation and soil moisture data are limited.

While the start date of pulse events is typically well-defined, determining the end date is more ambiguous, as the end date corresponds to the system either returning to prepulse conditions or reaching a stabilized state. However, since carbon emissions are greatest in the first few days of pulse events, potential misidentification of the end date is unlikely to impact overall CO₂ loss estimates. Future work should further optimize detection algorithms and test whether rain-induced CO_2_ pulses occur in other biomes whose high vegetation activity could mask their signals. Overall, the random forest gives insight into the drivers of pulse responses, enabling a deeper understanding of pulse responses in dryland ecosystems.

## Implications for terrestrial carbon cycle projections

Drylands drive the interannual variability and trend of global land carbon sink, but the processes underlying the rain-induced carbon losses responsible for carbon flux variability have not been widely incorporated in terrestrial ecosystem carbon models^60^. Our study demonstrates that rain-induced carbon losses follow a significant and consistent decay pattern across sites and contribute significantly to the carbon balance of drylands (16.9% ± 2.8% of annual ecosystem respiration and 9.6% ± 2.2% of annual NEP). We show that existing partitioning methods substantially underestimate carbon fluxes in drylands, potentially affecting higher-level ecosystem carbon models and upscaling studies that rely on flux partitioned data. We resolve persistent biases in current eddy-covariance partitioning methods by providing FluxPulse, a bias-corrected dataset of ecosystem carbon fluxes in drylands. Our results highlight the importance of effectively incorporating rain-induced carbon losses from pulse events into models from site to global scales. This is particularly important given the projected expansion of drylands in the twenty-first century^61^ and rapid changes in global precipitation patterns favouring more intense and less frequent rainfall^57^.

## Methods

### Data sources

We collected openly available eddy-covariance data from FLUXNET2015^62^, AmeriFlux (www.ameriflux.lbl.gov) and ICOS (Integrated Carbon Observation System) Warm Winter 2020 (ref. ^63^) (www.icos-cp.eu), all produced through ONEFlux processing under a CC-BY-4.0 license^62^. We used only original half-hourly measurements extracted from the gapfilled versions of NEE of CO_2_ (NEE_VUT_REF), latent heat flux (LE_F_MDS) and sensible heat flux (H_F_MDS), along with meteorological variables such as air temperature (TA_F_MDS) and incoming shortwave radiation (SW_IN_F_MDS). For precipitation, we gathered both tower-measured precipitation (*P*) and downscaled precipitation (P_ERA) from ERA-Interim reanalysis data products published via the above eddy-covariance networks^64^.

**Extended Data Fig. 1:**
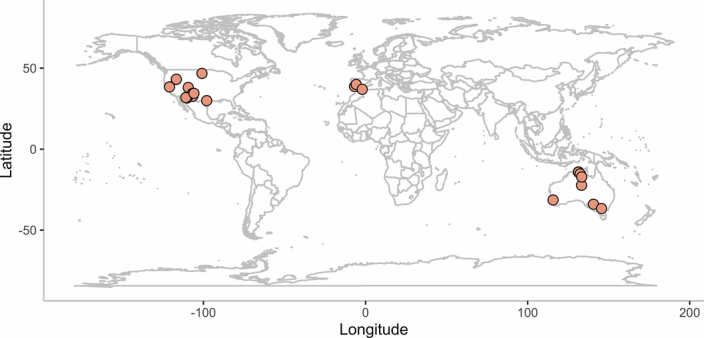
Global map of sites included in this research. Thirty-four eddy-covariance sites were drawn as orange points.

We selected eddy-covariance sites that satisfied the following criteria: (1) ratio of annual precipitation to potential evapotranspiration (*P*/PET) <0.65, a common definition of a dryland^65^, (2) at least 4 years of data and (3) short and sparse vegetation (typically height <2 m and area <30% of tree cover) with IGBP vegetation types belonging to grasslands, savannas, open shrublands, closed shrublands and woody savannas (WSA). We selected ecosystems with short and sparse vegetation to minimize rain loss from vegetation interception and reduce strong vegetation interference with rain-induced CO_2_ pulse signals detected in NEE measurements. In total, our data contained 34 sites across North America, Europe and Australia, with mean annual precipitation (MAP) ranging from 245 to 1,180 mm, mean annual temperature ranging from 7 °C to 29 °C and record length ranging from 4 to 21 years in each site (Extended Data Fig. 1 and Supplementary Table 1).

### Identification of pulse events

We established a foundational database of pulse events by manually labelling the start and end dates of these events, across 323 site-years of data in 34 sites. We then developed a machine learning algorithm to automatically detect pulse events from half-hourly eddy-covariance data, which was trained on the manually labelled dataset.

#### Pulse event indicator

**Extended Data Fig. 2:**
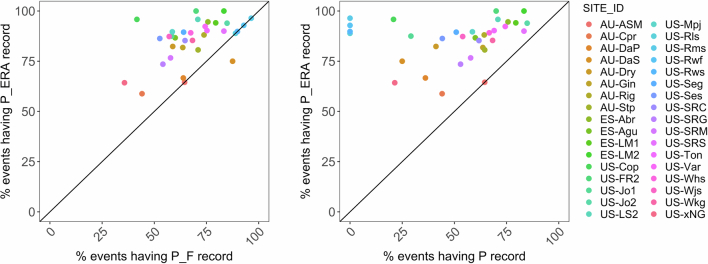
Percent of pulse events that have precipitation data record in 34 sites. Percent of pulse events that have precipitation data record (P, P_F, and P_ERA columns in FLUXNET database) within ±1 day from the start day of the events in 34 sites (0.001 mm was set as the threshold for precipitation occurrence). P_ERA aligned most accurately with the occurrence of pulse events, whereas P_F and P records were absent for several of these events.

**Extended Data Fig. 3:**
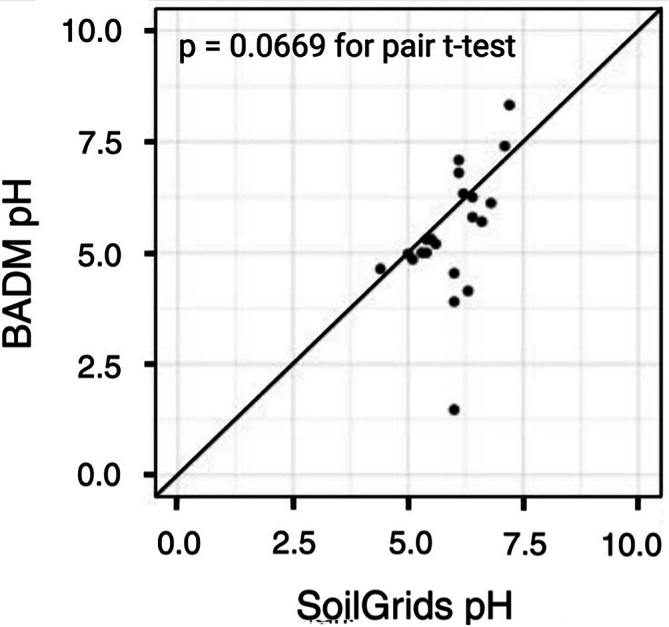
Comparison of SoilGrids soil pH and in-situ measurements. Soil pH obtained from Soilgrids was compared to soil pH obtained from the BADM (Biological, Ancillary, Disturbance and Metadata) of AmeriFlux & ICOS sites.

Rainfall is typically measured by the tipping bucket method in the eddy-covariance network; nevertheless, this method is known to under-report rainfall due to instrument limitations and landscape heterogeneity^58,59^. We confirmed that many tower data probably under-reported precipitation by noting that precipitation downscaled from ERA (P_ERA) is more synchronous with net CO_2_ pulses than the in situ-measured precipitation (*P*) (Extended Data Fig. 2). We therefore used P_ERA as the primary rainfall record.

In addition to rainfall, we used daytime EF as an index of soil water availability and rainfall occurrence. EF is the fraction of available energy allocated to evapotranspiration from the land surface, which we calculated on a daily scale as the average daytime ratio between latent heat flux and the sum of latent and sensible heat fluxes^66^. Daytime EF varies between 0 and 1, with 1 indicating a non-water-limiting condition as all available energy is converted to latent heat and 0 indicating a water-limited condition since all available energy is converted to sensible heat. EF range is 0.19 ± 0.18 to 0.52 ± 0.15 across 34 studied sites, including both growing and non-growing seasons. Rainfall after a long dry period creates a surge of EF, and dry ecosystems rarely get too wet for EF to saturate, making EF a potential indicator of soil water availability and rainfall occurrence. Compared with in situ soil water content measurements, *EF* is broadly available and can be calculated consistently across eddy-covariance sites.

#### Manual labelling of pulse events

We manually labelled the start and end dates of pulse events via observations of net CO_2_ pulses, aiming to study their spatiotemporal dynamics and to develop supervised machine learning models for labelling these events (Extended Data Fig. 10 for detailed criteria for labelling). We used original half-hourly time series data on NEE of CO_2_, daytime EF and downscaled precipitation (P_ERA) from ERA-Interim reanalysis data. Across 34 sites over 323 site-years, we manually labelled 1857 pulse events, with durations ranging from 2 to 26 days. The start date of the pulse event was labelled based on satisfying the following conditions: (1) NEE suddenly increases sharply, (2) NEE gradually decays over at least 2 days after the peak day to distinguish the start of pulses from random noises, (3) EF suddenly increases sharply and (4) positive precipitation (Supplementary Table 2). Sometimes, condition (4) was not satisfied, but we still marked the start of the pulse event because it is likely that rainfall was under-reported. The end date of the pulse event was defined as the date when night-time NEE stabilizes and approximates its prepulse magnitude (Extended Data Fig. 10).

#### Machine learning labelling of pulse events

We used random forests to automatically label pulse events at a half-hourly scale, since random forests or tree-based models in general, have been shown to outperform deep learning on tabular datasets^67^. Furthermore, random forest can inherently handle missing time series data and is more time-efficient compared with deep learning algorithms. Specifically, we applied the random forest binary classification algorithm using the function ‘RandomForestClassifier’ in the Python ‘sklearn’ package. The algorithm, trained on previously manually labelled datasets, processes FLUXNET formatted data to label measurements as pulse events or not on a half-hourly scale. To train and evaluate this model, we first selected 28 out of 34 sites that each has at least 5 years of data and 20 recorded pulse events. We trained one foundation model using data from all selected sites, using the last 3 years at each site as the testing set; the rest of the observations were randomly split into a training set and a validation set of ratio 80%:20%. This approach not only enhances the generalization capability of our model but also mitigates the risk of overfitting. The validation set was used for model selection purposes (such as tuning hyper-parameters), while the test set was used to evaluate the model’s final performance. The features used by the random forest model include hydrologic and carbon flux characteristics that are important signals for the pulse events, including rewetting intensity (ΔEF), antecedent water availability (prepulse EF), pulse intensity (ΔNEE) and antecedent ecosystem productivity (prepulse NEE) (Methods). The full list can be found in Fig. 4c.

We evaluate this model, again with respect to the test set, using two common metrics for classification tasks: precision and recall. Precision is the fraction of pulse events the model detected that were actually real $\left(\frac{{\rm{true}}\;{\rm{positive}}}{{{\rm{true}}\;{\rm{positive}}}+{{\rm{false}}\; {\rm{positive}}}}\right)$, while recall is the fraction of actual pulse events that were successfully detected by the model $\left(\frac{{\rm{true}}\;{\rm{positive}}}{{{\rm{true}}\; {\rm{positive}}}+{\rm{{false}}}\;{{{\mathrm{negative}}}}}\right)$. For example, if there are 12 pulse events in total, and the model detected 10 events with only 7 of them being actual pulse events, then precision is 7/10 and recall is 7/12.

Finally, the random forest model allowed us to investigate feature importance, that is, how relatively useful each covariate is in predicting the outcome. In particular, for each feature, we computed the normalized total reduction in the Gini impurity across decision tree leaves. Higher Gini importance indicates that the feature reduces more uncertainty in prediction on average and hence is more important^68^. We report these scores in Fig. 4c.

### Characterizing rain-induced carbon pulses

#### Length, intensity and size

We characterized rain-induced carbon pulses within the eddy-covariance network by defining the length, intensity and size of NEE during each event (equation (1) and Fig. 1). Even though the decay rate is an important feature of pulse events (Fig. 1), due to the uncertainties of half-hourly data and heterogeneity in data quality across sites, it is not possible to derive the decay rate for each event. Instead, we characterized the site-specific decay rate for all pulse events within one site (see ‘Site-specific pulse intensity and decay rate’). Regarding pulse length, we defined it as the number of days of the pulse events from the start to the end dates, which was directly calculated from the manually labelled dataset. Pulse intensity is, conceptually, the initial response of microbial respiration (*R*_h_) to rainfall, which was calculated as the change in NEE (ΔNEE) between the mean of 2 days after and the mean of 2 days before rainfall (Fig. 1). NEE was used instead of *R*_h_ to calculate the pulse intensity since vegetation has a lagged response to rainfall, hence ΔNEE is equal to Δ*R*_h_ on the first few days of the pulse events, and the eddy-covariance network does not provide direct measurements of *R*_h_ (Fig. 1). Regarding pulse size, we defined it as the sum of the daily mean of half-hourly NEE during the whole period of pulse events (Fig. 1).

#### Site-specific pulse intensity and decay rate

To estimate site-specific pulse intensity and decay rate, we fitted a first-order kinetic equation, which aims to mimic the litter decomposition function^50^

1 $${{{\mathrm{NEE}}}}_{{{i}},{{s}}}={\alpha }_{{{s}}}\times {{\mathrm{e}}}^{-{k}_{{{s}}}\times i},$$

where NEE_*i*,*s*_ is the maximum of daily mean NEE on day *i* from all pulse events in site *s* (equation (1)). *α*_*s*_and *k*_*s*_are the site-specific pulse intensity and decaying rate of site *s*, respectively (equation (1)). The site-specific pulse intensity *α*_*s*_is obtained by fitting equation (1) to all pulse events from one site, which is different from the pulse intensity of individual pulse events mentioned above (ΔNEE), even though they represent a similar concept of the initial response of the net carbon flux to rainfall.

### Existing *R*_eco_/GPP partitioning methods

#### Parametric methods

The partitioning methods we utilize partition *R*_eco_ and GPP from NEE based on the relationship NEE = *R*_eco_ – GPP, in which NEE is the NEE of CO_2_ fluxes measured directly from eddy-covariance towers, *R*_eco_ is ecosystem respiration and GPP is gross primary productivity (Fig. 1). We evaluated the performance of partitioning methods on estimating *R*_eco_ and GPP during pulse events and annually. The common parametric approaches that have been widely used by the flux community are the night-time method, which derives *R*_eco_ from night-time data as an Arrhenius-type function of temperature^34,69^, and the daytime approach, which models GPP and *R*_eco_ based on the light-response curve of GPP and temperature response of *R*_eco_(ref. ^42^). Both approaches model *R*_eco_ at half-hour *j* as an exponential function of air temperature (equation (2))^69^

2 $${\mathrm{{Rec}{o}}}_{{{\rm{NT}}}_{{\rm{method}}}{{\;j}}}={R}_{{\rm{ref}}}\times {{\mathrm{e}}}^{{E}_{0}\times \left(\frac{1}{{T}_{{{\mathrm{ref}}}}-{T}_{0}}-\frac{1}{{T_{\mathrm{air},\;j}}-{T}_{0}}\right)},$$

where ${\mathrm{{Rec}{o}}}_{{{\rm{NT}}}_{{\rm{method}}}{{\;j}}}$ is *R*_eco_ modelled by the night-time method at half-hour *j*, *T*_air,*j*_is the air temperature at half-hour *j*, *R*_ref_ is reference respiration at reference temperature (*T*_ref_ = 288.15 K) and *T*_0_ = 227.13 K and *E*_0_ is temperature sensitivity (Fig. 2c).

#### Machine learning methods

In addition to evaluating the parametric approaches, we assessed machine learning methods to partition *R*_eco_ and GPP during pulse events, which are a hybrid model based on artificial NN^44^ and a causal hybrid model based on DML^43^. The NN approach integrates physical constraints to simultaneously estimate *R*_eco_ and GPP through two sub-networks and showed improved flux estimates during pulse events at the US-SRG site^44^. The causal hybrid model enhances the hybrid model by incorporating principled causal knowledge and employs the DML approach for estimating causal effects^43^. Each model incorporates variables such as soil water content, shortwave radiation, day of the year, wind speed, vapour pressure deficit and air temperature as predictors. We reimplemented both approaches and ran them on our selected sites.

### FluxPulse, modelling rain-induced CO_2_ pulses from flux data

We developed the FluxPulse algorithm to model rain-induced carbon pulses from eddy-covariance flux data in 34 studied global dryland sites. FluxPulse bias-corrects *R*_eco_ and GPP from the night-time method during pulse events and prepulse periods, in which the night-time method fails to capture the temporal dynamics (Fig. 2c). For non-rewetting and non-prepulse periods, FluxPulse retains *R*_eco_ and GPP estimates from the NT method. A visualization tool for FluxPulse bias-corrected datasets can be accessed via ref. ^70^.

#### Bias-correct carbon fluxes during pulse events

To bias-correct both *R*_eco_ and GPP during pulse events, we first bias-corrected *R*_eco_, then calculated GPP as the difference between bias-corrected *R*_eco_ and NEE. In summary, FluxPulse re-estimates *R*_eco_ during pulse events by forcing *R*_eco_ on the first day of pulse events to approximate maximum NEE on the same day, then calculating *R*_eco_ in the following days using the decay rate *k*, which is computed empirically for each pulse event (equation (4)). A correction factor *β*, which was written as a decay function and calculated on a daily scale, was applied to all half-hourly night-time method *R*_eco_ ($\left.{{\mathrm{Reco}}}_{{{\rm{NT}}}_{{\rm{method}}}}\right)$) from the same day to obtain the half-hourly FluxPulse *R*_eco_ (${{\mathrm{Reco}}}_{{\rm{FluxPulse}}}$) (equation (3))

3 $${{\mathrm{Reco}}}_{{\rm{FluxPulse,}}{{i}},{\;{j}}}={\beta }_{{{i}}}\times {{\mathrm{Reco}}}_{{{\rm{NT}}}_{{\rm{method}}}{{i}},{\;{j}}},$$

where ${{\mathrm{Reco}}}_{{\rm{FluxPulse}},{i},{{\;j}}}$ is the FluxPulse bias-corrected *R*_eco_ on day *i* and half-hour *j*, and *β*_*i*_is the correction factor applied to all ${{\mathrm{Reco}}}_{{{\rm{NT}}}_{{\rm{method}}}{{i}},{{\;j}}}$ on day *i* and half-hour *j*, in which ${{\mathrm{Reco}}}_{{{\rm{NT}}}_{{\rm{method}}}{{i}},{{\;j}}}$ is *R*_eco_ obtained from the NT method. *β* decays over time to represent the decaying characteristics of *R*_eco_ during pulse events^13,15^ (equation (4) and Fig. 1) and is calculated as

4 $${\beta }_{{\rm{i}}}=\frac{{\mathrm{NEE}}_{{98}_{1}^{{\rm{th}}}}}{\mathrm{Reco}{}_{{{\rm{NT}}}_{{\rm{method}}}{98}_{1}^{{\rm{th}}}}}\times \mathrm{e}^{-k\times \left(i-1\right)},$$

where ${{\mathrm{NEE}}}_{{98}_{1}^{{\rm{th}}}}$ and ${\mathrm{Reco}}_{{{\rm{NT}}}_{{\rm{method}}}{98}_{1}^{{\rm{th}}}}$ are, respectively, the 98th percentile of half-hourly NEE and night-time method *R*_eco_ on day 1 of the pulse event (equation (4)). The 98th percentile values were chosen to select the maximum NEE and might-time method *R*_eco_ during pulse events. On day 1 (*i* = 1), *β*_*i*_is equal to the ratio between the 98th percentile NEE and the 98th percentile night-time method *R*_eco_ (equation (4)), hence for ${{\mathrm{Reco}}}_{{{\rm{NT}}}_{{\rm{method}}}{{i}},{{\;j}}}$ equals to ${{\mathrm{Reco}}}_{{{\rm{NT}}}_{{\rm{method}}}{98}_{1}^{{\rm{th}}}}$, ${{\mathrm{Reco}}}_{{\rm{FluxPulse}},{{i}},{{\;j}}}$ is equal to the 98th percentile NEE. This bias-correction method reduces the bias of the NT-method *R*_eco_ and forces the maximum of FluxPulse *R*_eco_ to approximate maximum NEE during pulse events, given *R*_eco_ should be theoretically larger or equal to NEE (equation (3)).

In equation (4), *k*is theoretically the decay rate of each pulse event. Since the half-hourly data are often noisy, and we do not have direct, continuous measurements of *R*_eco_ or *R*_h_, the decay rate *k* was estimated empirically as follows (Extended Data Figs. 5 and 6). First, we fitted a first-order kinetic equation similar to equation (1) to the array ${{\mathrm{NEE}}}_{{68}_{1}^{{\rm{th}}}}$, ${{{\mathrm{NEE}}}}_{{68}_{2}^{{\rm{th}}}}$, ${{{\mathrm{NEE}}}}_{{68}_{3}^{{\rm{th}}}}$… ${{{\mathrm{NEE}}}}_{{68}_{{\rm{i}}}^{{\rm{th}}}}$ in which ${{{\mathrm{NEE}}}}_{{{{p}}}_{{{i}}}^{{\rm{th}}}}$ is the *p*th percentile of all half-hourly NEE on day *i*. The 68th percentile was chosen to reflect values within ±1 standard deviation from the mean on each day. If the nonlinear fit is statistically significant (*P* < 0.1), then the estimate of *k* is accepted. If the nonlinear fit is not statistically significant, we fitted a first-order kinetic equation to an alternative array: $\,\bar{{{{\mathrm{NEE}}}}_{1}}$, $\bar{{{\mathrm{NEE}}}_{2}}$, $\bar{{{{\mathrm{NEE}}}}_{3}}$ … $\bar{{{{\mathrm{NEE}}}}_{i}}$, in which $\bar{{{{\mathrm{NEE}}}}_{i}}$ is the mean of all half-hourly NEE in day *i* from all pulse events in a specific site. We validated FluxPulse *R*_eco_ against NEE at night, which was assumed to approximate *R*_eco_ due to the lack of photosynthesis at night (Extended Data Figs. 5 and 6). We only selected pulse events that lasted longer than 3 days for validation to minimize the effect of random error and the potential introduction of uncertainties due to low-turbulence conditions.

It is worth noting that in equation (4), by using NEE to set FluxPulse *R*_eco_ during pulse events, we implicitly assume that GPP during the first day of rainfall is minimal due to the prepulse dry periods. Even though this is not the case in most sites (Extended Data Fig. 7), the assumption is necessary as there are no direct measurements of GPP and the partitioning methods available cannot provide reliable GPP estimates during pulse events. Importantly, this assumption is conservative and does not inflate the magnitude or importance of the main results. If GPP is positive on the first day of the pulse event, the bias in *R*_eco_ is expected to be even larger, as *R*_eco_ = NEE + GPP. Our reported bias values therefore probably underestimate the true bias associated with the partitioning methods assessed during pulse events (Fig. 2a,b).

#### Bias-correct carbon fluxes during prepulse periods

In addition to being biased during pulse events, the NT partitioning method is biased in the days leading up to pulse events (Fig. 2c and Extended Data Fig. 5). This is due to the fact that the NT method uses a moving window to estimate *R*_ref_ (equation (2)), and this window overlaps with the pulse event periods in the days before the pulse event. We therefore bias-corrected *R*_eco_ and GPP before the pulse events to reduce the prepulse biases (Fig. 2c, Extended Data Fig. 5 and Methods). We selected the prepulse period as *n* days before the pulse events (*n* being the event length) and used a 2-day window to estimate *R*_ref_ instead of a 4-day window used in the night-time method^34^ (equation (2)). Only prepulse periods that do not overlap with previous pulse events were selected for correction.

#### FluxPulse technical assessment

Validation against the NEE measurements at night is complicated by the fact that rain events greatly reduce data quality for eddy-covariance measurements, particularly for open-path sensors. The three sites in the database use enclosed CO_2_ sensors (ES-LM1, ES-LM2 and ES-Abr), however, which are robust under rainfall conditions^71^. Evaluation against night-time observations for those three sites shows that FluxPulse reduces the median *R*_eco_ bias from 27.0% to 0.7% (Extended Data Fig. 5). Across 82% of all studied sites, FluxPulse reduces biases significantly from the night-time method (*P* < 0.001, one-tailed paired *t*-test) and successfully captures the pulse temporal dynamics (Extended Data Figs. 5 and 6).

### Hydrologic characteristics

**Extended Data Fig. 4:**
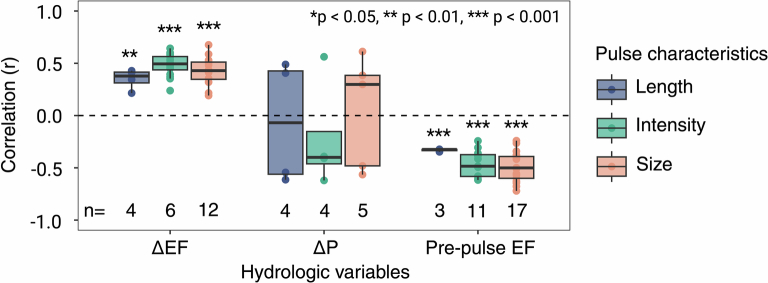
Pearson correlation between pulse characteristics (Length, intensity, and size) and hydrologic variables (rewetting intensity (ΔEF, ΔP), antecedent water availability (Pre-pulse EF)) for each site. Only sites with a significant linear relationship with climatic drivers are shown as points (p *<* 0.05). Asterisks indicate the level of two-tailed t-test significance for whether the mean of the distribution is different than 0. The boxes represent interquartile range (IQR) marked with 25^th^, 50^th^, 75^th^ percentile and whiskers extending to 1.5 times the IQR. n indicates the number of points in each boxplot.

**Extended Data Fig. 5:**
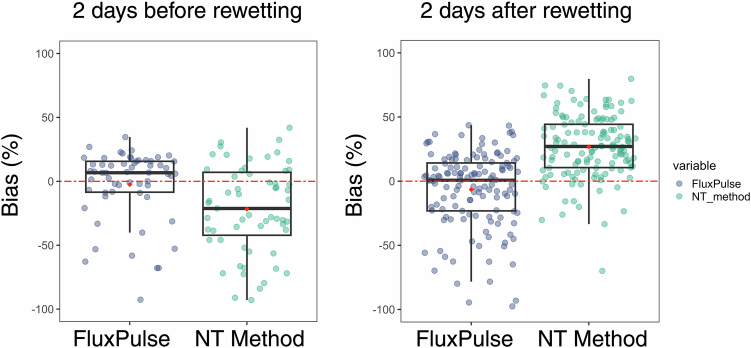
Bias (%) of FluxPulse *R*_eco_ and Nighttime (NT)-method *R*_eco_ when comparing to nighttime *NEE* during two days before and after the start of pulse events at three sites that use an enclosed-path system (ES-LM1, ES-LM2, and ES-Abr). Bias (%) is calculated as (*x*-*y*)/x*100%, in which *x* is the 68^th^ percentile (1 standard deviation) of half-hourly Nighttime *NEE*, and y is the 68^th^ percentile of half-hourly modelled *R*_eco_ (either FluxPulse or NT-method *R*_eco_) during two days before and after the start of pulse events. Note that there are fewer points before pulse events than after because we only bias-corrected the pre-pulse periods that are not overlapped with previous pulse events. The pre-pulse period covers *n* days before each pulse event, in which *n* is the length of that pulse event. The boxes represent interquartile range (IQR*)* marked with 25^th^, 50^th^, 75^th^ percentile and whiskers extending to 1.5 times the IQR. The red diamonds represent the mean of the boxplots.

**Extended Data Fig. 6:**
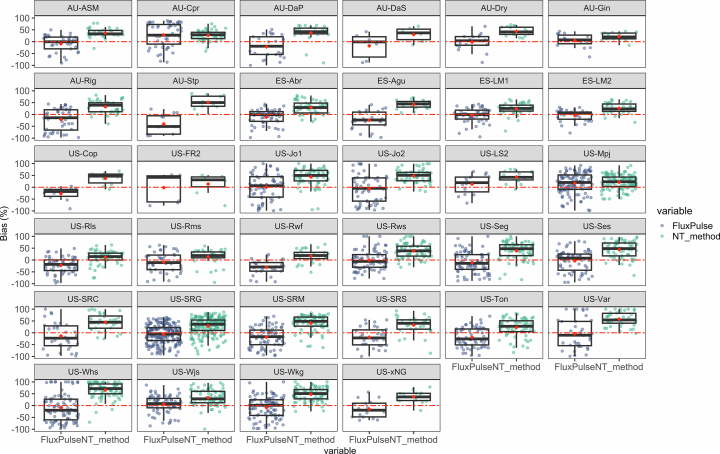
Bias (%) of FluxPulse R_eco_ and Nighttime (NT)-method R_eco_ when comparing to nighttime NEE during two days after the start of pulse events at 34 sites. Bias (%) is calculated as (x-y)/x*100, in which x is the 68^th^ percentile (1 standard deviation) of half-hourly Nighttime NEE, and y is the 68^th^ percentile of half-hourly modelled R_eco_ (either FluxPulse or NT-method R_eco_) during two days after the start of pulse events. The boxes represent interquartile range (IQR) marked with 25^th^, 50^th^, 75^th^ percentile and whiskers extending to 1.5 times the IQR. The red diamonds represent the mean of the boxplots.

**Extended Data Fig. 7:**
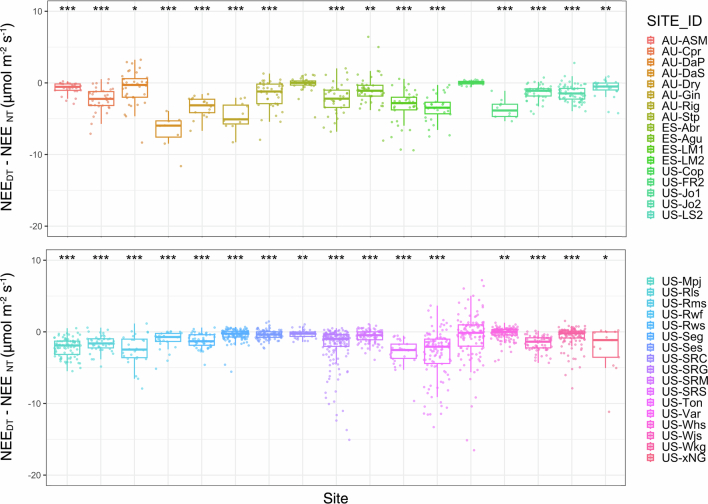
Difference between half-hourly *NEE* during daytime and nighttime for the period of two days before each pulse event. Difference between half-hourly *NEE* during daytime and nighttime (*NEE*_DT_ - *NEE*_NT_) for the period of two days before each pulse event. *NEE*_DT_ - *NEE*_NT_ could indicate the presence of vegetation activity before pulse events, since the more negative is *NEE*_DT_ – *NEE*_NT_, the more likely vegetation is active (*NEE*_DT_ - *NEE*_NT_ = (*R*_eco DT_ – *GPP*) – *R*_eco NT_ = (*R*_eco DT_ – *R*_eco NT_) – *GPP*). Assuming that *R*_eco DT_ – *R*_eco NT_ is positive since *R*_eco_ increases with temperature, then the observed negative *NEE*_DT_ - *NEE*_NT_ must be due to positive *GPP*. Asterisks indicate the level of one-tailed t-test significance for whether the mean of the distribution is greater than 0 (*p < 0.05, **p < 0.01, ***p < 0.001). The boxes represent interquartile range (IQR*)* marked with 25^th^, 50^th^, 75^th^ percentile and whiskers extending to 1.5 times the IQR.

Carbon flux characteristics during pulse events are strongly affected by the level of soil dryness and rainfall rewetting intensities^14,15^. We tested two different indices for rewetting intensity: the EF and precipitation (*P*) (Extended Data Fig. 4). The rewetting intensity (ΔEF or Δ*P*) is the difference in either EF or *P* between 2 days after and 2 days before the rainfall. We calculated the antecedent water availability, denoted as prepulse EF, as the mean of EF 15 days before rainfall. A lower prepulse EF indicates a drier antecedent period. We took the average EF in 15 days before the pulse events happened but not a longer or shorter period, because a longer time window could include a mix of growing season and non-growing season, and a shorter period does not fully represent the history of water stress.

### Soil characteristics

Microbial response to rewetting is influenced not only by water dynamics but also by soil properties^15,72^. We extracted soil chemical and physical properties such as soil pH, texture and SOC from SoilGrids (www.soilgrids.org), which is a 250-m resolution interpolated global map of soil properties^73^.

### Analysis of spatiotemporal drivers of rain-induced carbon losses

We used the Lindeman, Merenda and Gold (LMG) method in the R package ‘relaimpo’ to assess the importance of spatial drivers such as soil characteristics, vegetation uptake, and climatic conditions on the intensity and decay rate of NEE during pulse events (Fig. 3). The LMG method is robust to multicollinearity between variables and provides confidence interval estimation via bootstrapping^74^. Regarding the temporal drivers of rain-induced carbon losses, we used the feature importance scores obtained from using random forests to label pulse events (‘Identification of pulse events’ section).

**Extended Data Fig. 8:**
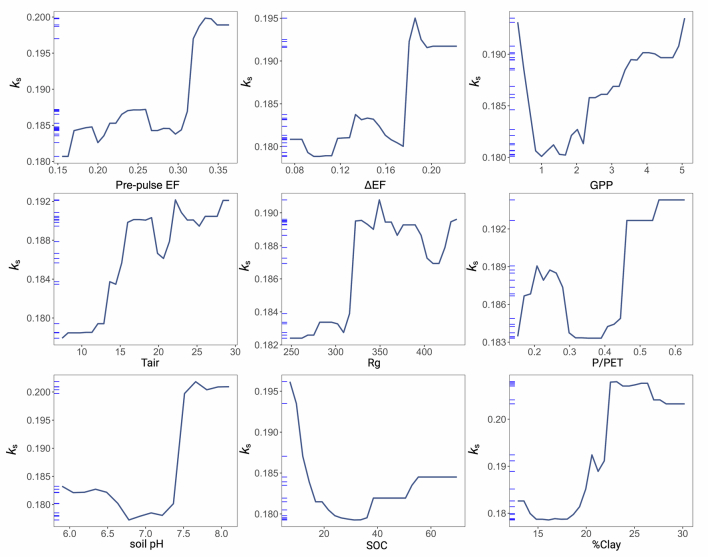
Partial Dependence Plots for the responses of site-specific decay rate to nine predictors. Partial Dependence Plots for the responses of site-specific decay rate (*k*_s_) to nine predictors. Predictors related to soil characteristics are clay percentage (*%Clay*), soil organic carbon from 0-5 cm (*SOC*) (g kg^−1^), and soil *pH*. The predictor representing vegetation productivity is annual *GPP* obtained from the Nighttime (NT) partitioning method (µmol CO_2_ m^−2^ s^−1^). Predictors representing hydrologic conditions are aridity index (*P/PET*), rewetting intensity (*ΔEF*), and antecedent water availability (Pre-pulse *EF*). Predictors representing climatic conditions are global shortwave radiation (R_g_) (W m^−2^), and air temperature (*T*_air_) (^o^C).

**Extended Data Fig. 9:**
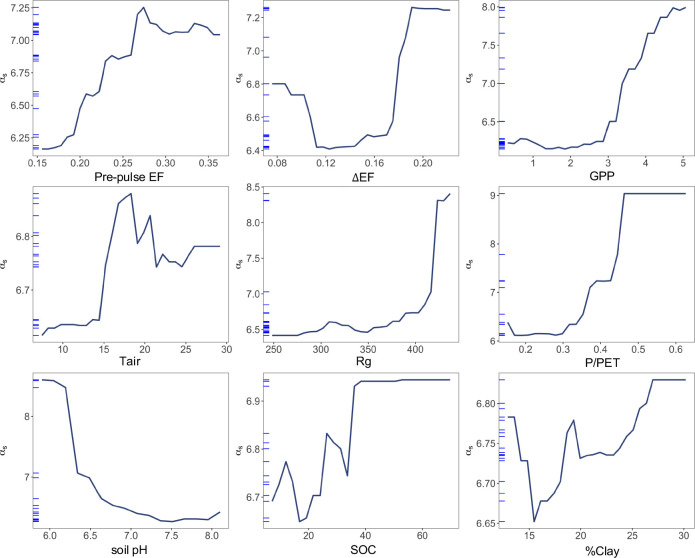
Partial Dependence Plots for the responses of site-specific pulse intensity to nine predictors. Partial Dependence Plots for the responses of site-specific pulse intensity (*α*_s_) to nine predictors. Predictors related to soil characteristics are clay percentage (*%Clay*), soil organic carbon from 0-5 cm (*SOC*) (g kg^−1^), and soil *pH*. The predictor representing vegetation productivity is annual *GPP* obtained from the Nighttime (NT) partitioning method (µmol CO_2_ m^−2^ s^−1^). Predictors representing hydrologic conditions are aridity index (*P/PET*), rewetting intensity (*ΔEF*), and antecedent water availability (Pre-pulse *EF*). Predictors representing climatic conditions are global shortwave radiation (*R*_g_) (W m^−2^), and air temperature (*T*_air_) (^o^C).

**Extended Data Fig. 10:**
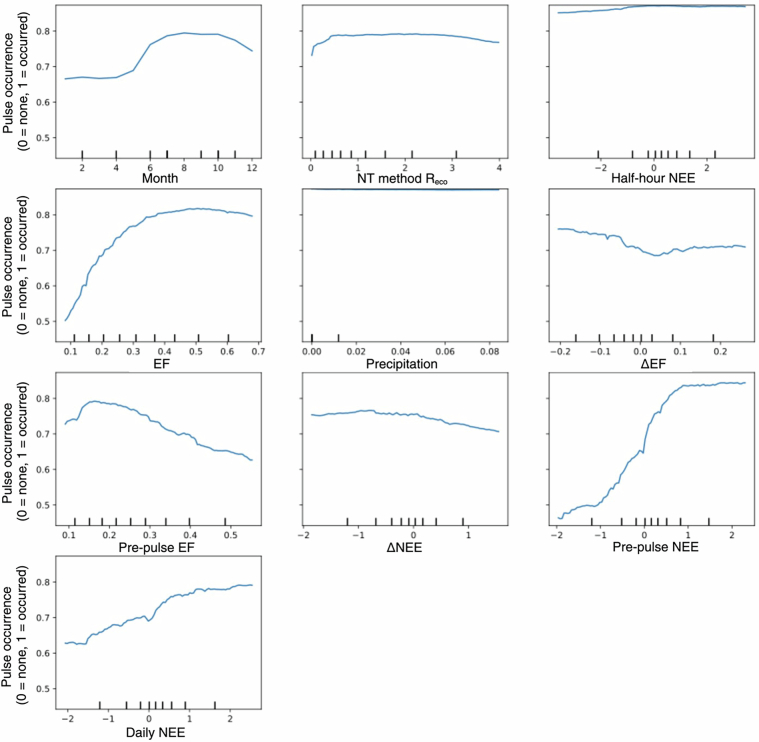
Partial Dependence Plots for the responses of pulse occurrence to temporal predictors. Partial Dependence Plots for the responses of pulse occurrence (value of 1 indicates that a pulse event occurred, while 0 represents no likelihood of a pulse event) to temporal predictors (see Methods and Fig. 4c).

To analyse the directional relationship between site-specific pulse intensity/decay rate and each predictor, we used partial dependence plot, which effectively considers confounding effects between multiple predictors and the target variable, without assuming a linear relationship such as the Pearson correlation (Extended Data Figs. 8 and 10).

## Online content

Any methods, additional references, Nature Portfolio reporting summaries, source data, extended data, supplementary information, acknowledgements, peer review information; details of author contributions and competing interests; and statements of data and code availability are available at 10.1038/s41561-025-01754-9.

## Supplementary information

Supplementary Tables Supplementary Table 1. List of sites included in this research and random forest performance on detecting the start date of pulse events in each site (within ±2 days error). Supplementary Table 2. The detailed criteria for manually labelling pulse events.

## Source data

Source Data Statistical source data for Figs. 2a–4c.

## Data Availability

Eddy-covariance sites are obtained from FLUXNET (www.fluxnet.org), AmeriFlux (www.ameriflux.lbl.gov) and ICOS (Integrated Carbon Observation System) Warm Winter 2020 (www.icos-cp.eu). Soil properties are obtained from Soilgrids (www.soilgrids.org). The manual pulse labelling dataset, FluxPulse bias-corrected eddy-covariance datasets, and datasets used to generate the figures are available via GitHub at https://github.com/ngocnguyen99/FluxPulse. Source data are provided with this paper.

## Code availability

Related codes are publicly available via GitHub at https://github.com/ngocnguyen99/FluxPulse. The FluxPulse visualization tool is available via Shiny Applications at https://ngoc-nguyen-ucberkeley.shinyapps.io/fluxpulse_visualization/.
